# Accupuncture-like transcutaneous electrical nerve stimulation therapy success using a commercially available unit 8 years post-radiation for xerostomia: a case report

**DOI:** 10.1017/S1460396917000024

**Published:** 2017-05-08

**Authors:** Austin J. Iovoli, Anurag K. Singh

**Affiliations:** Department of Radiation Medicine, Roswell Park Cancer Institute, Buffalo, NY, USA

**Keywords:** Acupuncture-like transcutaneous electrical nerve stimulation, ALTENS, quality of life, radiation, xerostomia

## Abstract

**Background:**

Accupuncture-like transcutaneous electrical nerve stimulation (ALTENS) therapy has been shown in prospective studies to be effective in the treatment of radiation-induced xerostomia. Those studies treated patients within 27 months from end of radiation with ALTENS delivered in the clinic using a Codetron unit. However, that unit is no longer produced and there is limited data on success of ALTENS when delivered at home.

**Methods:**

A 50-year old man with xerostomia, 8 years post-radiation for T4N1 squamous cell carcinoma of the tonsillar fossa, was given ALTENS with a currently commercially available unit from Girish Surgical. He used the unit at home, 20 minutes daily for 8 weeks.

**Results:**

After 8 weeks of ALTENS therapy the patient saw a reduction in the Self-Reported University of Michigan Xerostomia-Related Quality of Life Scale from 20 to 1.

**Conclusion:**

This case report demonstrates (1) the Girish Surgical unit is effective, (2) self-administration of ALTENS in patients who cannot come to clinic regularly may be practical and (3) ALTENS can still offer durable benefit to patients even 8 years after chemoradiation therapy.

## INTRODUCTION

Xerostomia is a common toxicity associated with chemoradiation in the treatment of head and neck cancer. This occurs in the vast majority of patients treated with radiation to the head and neck as a result of damage to the salivary glands, causing hyposalivation and chronic oral dryness. Patients with xerostomia are at higher risk of oral infections and frequently experience problems with eating, speaking and swallowing.^1^ These effects are associated with an appreciable decrease in quality of life and long-term morbidity. Currently approved treatment options are limited and not always effective. The most commonly used therapy is pilocarpine, which increases salivary flow in the major salivary glands by stimulating M3 muscarinic receptors, but is associated with significant adverse side effects.^2,3^

**Figure 1:**
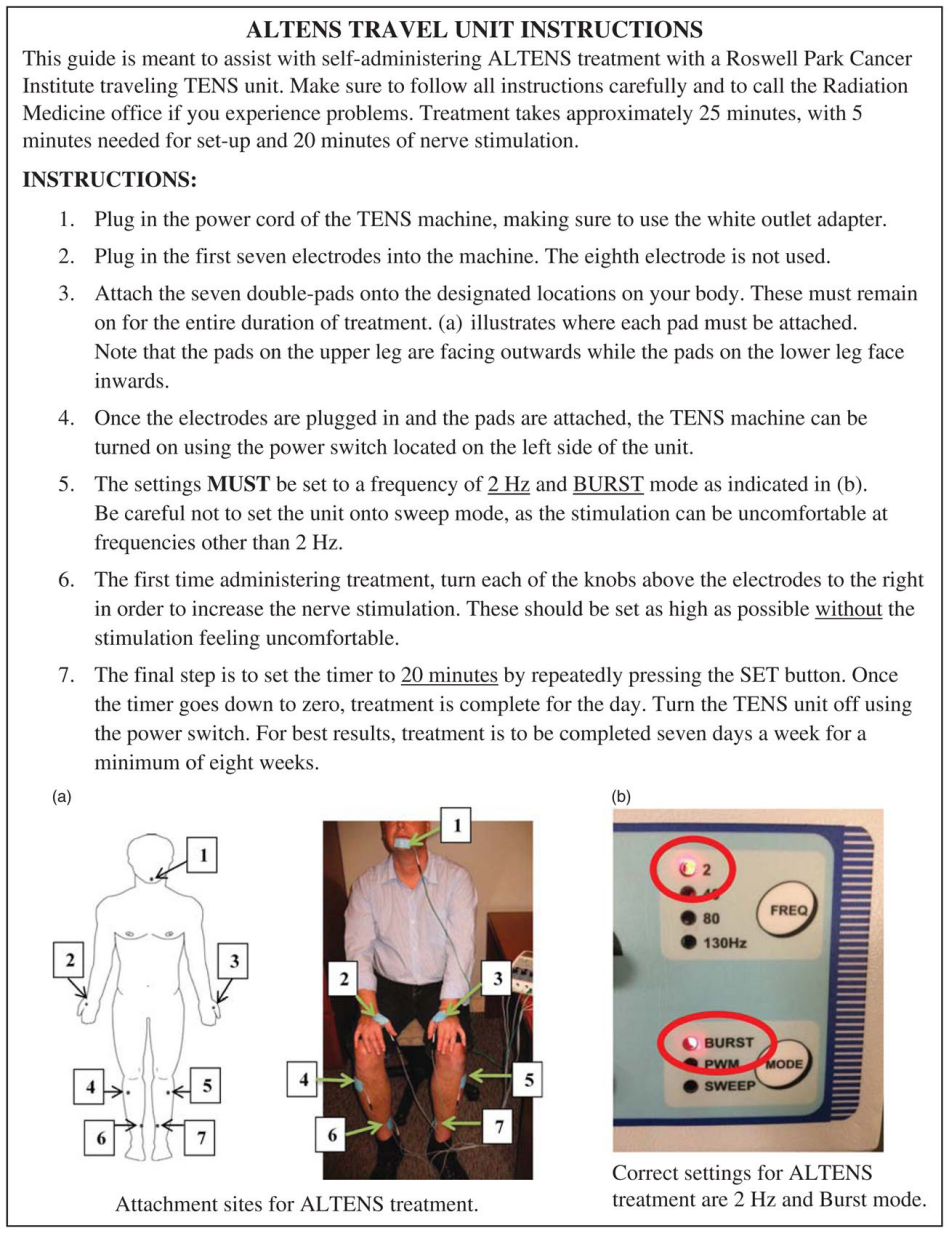
Patient accupuncture-like transcutaneous electrical nerve stimulation (ALTENS) self-administration instructions.

Acupuncture-like transcutaneous electrical nerve stimulation (ALTENS) treatment has emerged as a promising new modality to improve saliva production and related symptoms without the associated cholinergic side effects caused by sympathomimetic agents.^4–7^ Reports generated from single and multi-institutional studies have demonstrated benefit in patients initiating ALTENS therapy within 3–27 months of completing chemoradiation.^4–7^ In addition, a previous case report found ALTENS therapy to be effective in a patient 5 years removed from completion of chemoradiation.^8^ Unfortunately, the TENS unit produced by Codetron (Buffalo, NY, USA) used to conduct these studies is no longer manufactured. The treatment protocol for these patients also required them to receive therapy at a clinical centre twice weekly for 12 weeks.^4–7^ A different TENS unit, produced by Girish Surgical (Mumbai, India) is available and in current production. This unit was purchased, instruction on its use was given to a patient in the clinic (Figure 1), and the unit was loaned to the patient for self-use at home. Self-administrated ALTENS therapy using a portable Girish Surgical TENS unit has not previously been evaluated.

## CLINICAL HISTORY

A 50-year old male with a history of T4N1M0 squamous cell carcinoma of the tonsillar fossa underwent definitive treatment with chemoradiation. Radiation was delivered from 20 August 2007 through 8 October 2007 to the oropharynx, entire neck and supraclavicular region using intensity-modulated radiation therapy. A total dose of 7,500 cGy was delivered in 125 cGy fractions given twice daily, 5 days per week. Overall treatment time for these 60 fractions was 51 days. The patient tolerated treatment relatively well, however he developed trouble swallowing and xerostomia that persisted post-radiation. These toxicities did not improve over the next 8 years of follow up.

ALTENS therapy is available to all patients free of charge at Roswell Park Cancer Institute (RPCI). This involves using a Codetron eight-channel TENS unit and requires the patient to travel to RPCI twice a week to receive therapy. As this patient lives over an hour from Buffalo and Codetron TENS units are no longer produced in the United States, an eight-channel TENS unit was purchased from Girish Surgical in India. The patient was taught how to self-administer ALTENS and then given the unit to carry out therapy at home for the duration of treatment. A single round of daily treatment was self-administered for 8 weeks totalling 56 applications. Self-reported University of Michigan Xerostomia-Related Quality of Life Scale (XeQOLS) surveys^8^ were given at baseline and upon completion at 8 weeks to assess response and saw a reduction in score from 20 to 1. In addition, the patient reported improvements in salivary function and related symptoms that included ‘being moist more frequently, having less food stuck to teeth, and less dryness upon waking up from sleep’. These benefits have been maintained since completion of therapy 6 months ago.

## DISCUSSION

A series of studies have suggested that ALTENS may improve radiation-induced xerostomia. Wong et al.^4^ conducted a 46 patient, single institution, phase I–II trial demonstrating an ALTENS approach without invasive needles that improved whole saliva production and related symptoms in patients when initiated within 27 months of chemoradiation completion. The multi-institutional, 48 patient, phase II trial that followed showed similar efficacy with a positive treatment response in 86% of patients using the self-reported XeQOLS surveys to evaluate response at baseline and 6 months post-treatment.^5^ Eligibility for ALTENS was limited to those within 24 months of completing chemoradiation.^5^

In a 148 patient, phase III multi-institutional randomised study comparing ALTENS with pilocarpine therapy, Wong et al.^6^ found ALTENS was just as efficacious as pilocarpine but produced significantly less grade 3 or less adverse events (20·8–61·6%). A secondary analysis of this study using the RTOG-modified University of Washington Head and Neck Symptom Score to assess overall health-related quality of life demonstrated consistently lower scores, indicating better function, for patients randomised to the ALTENS arm compared with those randomised to pilocarpine.^7^

Since the publication of these studies, 1 case report has reported the effectiveness of initiating ALTENS in a patient 5 years removed from completing chemoradiation.^9^ With the Codetron TENS unit no longer in production, it became necessary to find an alternative 8-channel TENS unit to be used with ALTENS therapy. There have been no current or previous studies evaluating the effectiveness of a Girish Surgical TENS unit with ALTENS therapy or examining the efficacy of having a patient self-administer treatment with a travelling unit.

As a result of these clinical studies of ALTENS and the current report, it may be of value to continue evaluating the effectiveness of the Girish Surgical unit as a suitable replacement for the Codetron unit and the efficacy of ALTENS therapy in patients suffering from radiation-induced xerostomia who have been post-chemoradiation for an extended period of time. In addition, the possibility of allowing patients to self-administer treatment can be a practical solution to expanding ALTENS therapy to patients unable to come to clinic regularly. In this patient, we observed a compelling drop in XeQOLS score from 20 to 1 and the patient self-reported a noticeable improvement in symptoms. The Girish Surgical unit offers a promising replacement to the Codetron unit and has the potential to alleviate xerostomia symptoms in those well beyond post-chemoradiation completion.

## CONCLUSIONS

ALTENS has proven to be effective in alleviating symptoms of radiation-induced xerostomia, however the Codetron TENS unit used in these studies is no longer available. Our findings suggest the Girish Surgical TENS unit is effective, self-administration of ALTENS in patients who cannot come to clinic regularly may be practical, and ALTENS can still offer durable benefit to patients even 8 years after chemoradiation completion. Self-administration of ALTENS has the further advantage that patients may continue it until they are satisfied with the response as opposed to some arbitrary limit on the number of treatments.
